# Digitalisierung: Welche Rolle spielen CIOs heute und in Zukunft?

**DOI:** 10.1365/s40702-022-00868-7

**Published:** 2022-04-12

**Authors:** Walter Brenner, Barbara Brenner

**Affiliations:** Institute of Information Management, University of St. Gallen, St. Gallen, Schweiz

**Keywords:** Chief Information Officer, CIO, IT, IT-Strategie, Informationsmanagement, IT-Kultur, Chief Information Officer, CIO, IT, IT-Strategy, Information Management, IT-Culture

## Abstract

Viele CIOs (Chief Information Officer) in Unternehmen haben in der Pandemie einen hervorragenden Job gemacht. In der Zeit nach der Pandemie gilt es, diesen „Rückenwind“ und die Position des CIO neu zu definieren. Vor diesem Hintergrund wurden fünf CIOs, Hanna Hennig von der Siemens AG, Andreas Maier von der AXA Schweiz, Michael Müller-Wünsch von OTTO, Rolf Olmesdahl, ehemals Raiffeisen Schweiz, Ursula Soritsch-Renier von Saint-Gobain Paris, und ein Executive-Search-Spezialist, Patrick Naef, Boyden AG, gefragt wie sie die Zukunft des CIO sehen. Alle sechs Persönlichkeiten, die an dem Dialog teilnahmen, sind übereinstimmend der Meinung, dass es auch noch 2030 CIOs geben wird und sie auf der einen Seite verstärkt Treiber der digitalen Transformation in ihrem Unternehmen sein sollten und sie auf der anderen Seite nach wie vor Verantwortung für das Funktionieren der digitalen Infrastruktur tragen.

## Abstract (en)

Many CIOs (Chief Information Officers) in companies did an excellent job during the pandemic. In the post-pandemic period, it is important to redefine the position of the CIO. Five CIOs, Hanna Hennig of Siemens AG, Andreas Maier of AXA Switzerland, Michael Müller-Wünsch of OTTO, Rolf Olmesdahl, formerly of Raiffeisen Switzerland, Ursula Soritsch-Renier of Saint-Gobain Paris, and an executive search specialist, Patrick Naef, Boyden AG, were asked how they see the future of the CIO. All six executives who took part in the dialog agree that CIOs will still exist in 2030 and that, on the one hand, they should increasingly be drivers of the digital transformation in their company and, on the other hand, they still bear responsibility for the functioning of the digital infrastructure.

## Prolog

Zahlreiche Unternehmen und öffentliche Verwaltungen im deutschsprachigen Raum werden Chancen verpassen und dauerhaft Schaden erleiden, wenn sie nicht die Intensität und Umsetzungsgeschwindigkeit der Digitalisierung erhöhen. Digitalisierung wird als umfassender Einsatz der Informations- und Kommunikationstechnik in Unternehmen oder öffentlichen Verwaltungen, zur Automatisierung von Prozessen, zur Digitalisierung von Produkten und Dienstleistungen, zur Entwicklung neuer digitaler Produkte, Dienstleistungen oder Geschäftsmodelle verstanden (Leimeister et al. 2014). Vor diesem Hintergrund stellt sich für Unternehmen und öffentliche Verwaltungen die Frage, wer für Digitalisierung verantwortlich ist. Traditionell war es die Aufgabe der CIOs, sich um Informations- und Kommunikationstechnik und ihren Einsatz im Unternehmen zu kümmern. In den letzten zehn Jahren ist diese traditionelle Aufgabe der CIOs mehr und mehr bestritten worden. Die Digitalisierung von Produkten, beispielsweise Fahrzeugen, wurde von Forschungs- und Entwicklungsabteilungen verantwortet (Herrmann und Brenner 2018), für Data Analytics (Dremel et al. 2017; Bean 2020) gibt es eigene Bereiche und für die Digitalisierung des gesamten Unternehmens (Berkmann 2013; Singh und Hess 2017) wurde die Position des Chief Digital Officer geschaffen (Tumbas et al. 2018). Zudem gibt es inzwischen viele CEOs und Führungskräfte auf Ebene der Geschäftsleitung, die für sich die volle Verantwortung für Digitalisierung reklamieren (Lamarre et al. 2021). Trotz dieser Diskussionen in Unternehmen und der Wissenschaft haben nach wie vor fast alle Unternehmen einen CIO und eine IT-Abteilung.

In der Wirtschaftsinformatik ist die Beschäftigung mit dem CIO und der Führung der IT in den letzten Jahren etwas in den Hintergrund getreten (Gerster 2017). Es gibt einige wenige Artikel im Basket of Eight^1^, die sich beispielweise mit Informatikstrategien beschäftigen (Chen et al. 2010), mit Bimodaler IT (Haffke et al. 2017), der Zukunft des CIO in einer digitalen Wirtschaft (Weill und Woerner 2013), dem Beitrag des CIO zu Innovation (Chen et al. 2021), mit der strukturellen formalen Macht des CIO und den Auswirkungen auf die Performance eines Unternehmens (Feng et al. 2021), dem Einfluss von Sozialkapital auf die Beziehungen zwischen dem CIO und der Geschäftsleitung und den Auswirkungen auf die Performance des Unternehmens (Karahanna und Preston 2013), dem Ausgleich zwischen Nachfrage und dem Angebot an IT im Unternehmen (Chen et al. 2010), dem Einsatz Künstlicher Intelligenz (Li et al. 2021) und der Motivation für die Aufnahme des CIO in die Geschäftsleitung, um in diesem Gremium mehr technische Kompetenz im Sinne von Dynamic Capabilities aufzubauen (Bendig et al. 2022).

Aus Sicht der Autorin und des Autors dieses Beitrages lohnt es sich nicht nur aufgrund der geringen Anzahl wissenschaftlicher Beiträge in den führenden wissenschaftlichen Zeitschriften, die sich mit CIOs auseinandersetzen, sondern auch wegen der steigenden Bedeutung der Digitalisierung in der Pandemie, sich verstärkt mit den CIOs und ihrer Zukunft zu befassen. Vor diesem Hintergrund beschäftigt sich dieser Beitrag mit drei Forschungsfragen: (1) Was haben Unternehmen im Rahmen der Digitalisierung erreicht, (2) welchen Beitrag leisten die CIOs zur Digitalisierung und (3) mit welchen Herausforderungen werden sich CIOs in Zukunft auseinandersetzen müssen. Die Forschungsfragen werden im Rahmen eines Dialogs bearbeitet. Fünf CIOs aus Deutschland, Frankreich und der Schweiz sowie ein auf die Suche nach CIOs spezialisierter Personalberater kommen in dem Beitrag zu Wort. Die Leserinnen und die Leser können sich ein eigenständiges Bild machen, welche Antworten prominente CIOs auf die drei Forschungsfragen haben. Aufgrund der Pandemie und des engen Zeitrahmens konnten die CIOs nicht zu einem physischen Gespräch zusammenkommen und auch eine gemeinsame Videokonferenz war aus Termingründen unmöglich. Mit vier Persönlichkeiten fanden in einer ersten Runde ca. 90-minütige Gespräche statt. Zwei CIOs, Rolf Olmesdahl und Andreas Maier, haben aus Termingründen die Fragen schriftlich beantwortet. Tab. 1 zeigt, welche CIOs beteiligt waren und wann die entscheidenden ersten Gespräche stattfanden, bzw. wann die entscheidenden Antworten schriftlich eintrafen.

**Tab. 1:** Beteiligte CIOs und Termin des ersten Gesprächs

Name CIO	Unternehmen	Land	Termin Runde 1
Hanna Hennig	Siemens AG	Deutschland	25.11.2021
Andreas Maier	AXA Schweiz	Schweiz	26.11.2021
Michael Müller Wünsch	OTTO	Deutschland	29.11.2021
Patrick Naef	Boyden AG	Schweiz	18.11.2021
Rolf Olmesdahl	Ehem. Raiffeisen Schweiz	Schweiz	10.11.2021
Ursula Soritsch-Renier	Saint-Gobain Paris	Frankreich	24.11.2021

Für den Dialog mit den CIOs und dem Personalberater wurde derselbe Gesprächsleitfaden verwendet. Aus diesen Einzelgesprächen und schriftlichen Antworten wurde eine erste Version des Dialogs erstellt. Diese erste Version wurde allen CIOs zugestellt und sie konnten sehen, was ihre Kolleginnen und Kollegen geantwortet haben, konnten ihre Aussagen anpassen und ihren Presseabteilungen vorlegen. Nach dieser zweiten Runde ist die endgültige Version des Dialogs entstanden.

## Dialog mit Hanna Hennig, Siemens AG, Andreas Maier, AXA Schweiz, Michael Müller-Wünsch, OTTO, Rolf Olmesdahl, ehemals Raiffeisen Schweiz, Ursula Soritsch-Renier, Saint-Gobain, Paris, und Patrick Naef, Boyden AG

### *Walter Brenner:*

*Stellen Sie sich bitte kurz vor und beschreiben Sie Ihr Unternehmen, Ihre Funktion, für was Sie primär verantwortlich sind und, wenn Sie möchten, auch wie groß der Bereich ist, dem Sie vorstehen, und wie hoch Ihr Budget ist.*

### Hanna Hennig:

Seit Januar 2020 verantworte ich als CIO die IT für ca. 300.000 Mitarbeitende der Siemens AG, die in über 200 Ländern arbeiten. Siemens ist ein führendes Technologieunternehmen in den Bereichen Industrie, Infrastruktur, Mobilität und Gesundheit. Durch die Kombination der realen und der digitalen Welten ermöglichen wir unseren Kundinnen und Kunden ihre Industrien und Märkte zu transformieren und echte Mehrwerte zu schaffen. Damit verbessern wir den Alltag für Milliarden von Menschen und helfen die Herausforderungen in Umweltschutz, Dekarbonisierung, Gesundheit und Sicherheit zu bewältigen. Im Geschäftsjahr 2021 erzielte der Siemens-Konzern einen Umsatz von 62,3 Mrd. € und einen Gewinn nach Steuern von 6,7 Mrd. €. Unsere IT-Funktion führt die IT- und OT-Konvergenz im Unternehmen an und treibt die interne Digitalisierung unseres Unternehmens inklusive unserer Werke mit unseren Fachbereichen voran. Wir unterstützen bei der Entwicklung von Produkten und Geschäftsmodellen, sorgen für einen reibungslosen Ablauf der Geschäftsprozesse des Unternehmens und für ein exzellentes Nutzererlebnis mit Office- und Produktivitätstools.

### Andreas Maier:

Ich bin CIO und Mitglied der Geschäftsleitung der AXA Schweiz. Der IT-Bereich der AXA hat ca. 700 Mitarbeitende und ein Budget von ca. 210 Mio. Schweizer Franken. Nach meinem Fachhochschulabschluss in Informatik und einem Abschluss als Executive Master in Business Administration arbeitete ich drei Jahre als Head of Operations Europe bei der Winterthur Versicherungen Life & Pension Europe, bevor ich 2003 zu Zurich Financial Services wechselte. Dort war ich zuerst tätig als CIO im Global Life Business Segment, dann als CIO von North America Commercial Business und zuletzt als CIO im General Insurance Business Segment. Seit Juni 2012 bin ich Mitglied der Geschäftsleitung AXA Schweiz und Leiter der IT. Zusätzlich bin ich seit dem 1. April 2018 Verwaltungsratsmitglied von Sobrado und seit dem 11. Oktober 2021 Verwaltungsratspräsident der neu gegründeten InsureTech noimos AG.

### Michael Müller-Wünsch:

Ich bin ein in Hamburg lebender Berliner mit Informatik-Abschluss und Promotion im Bereich der Künstlichen Intelligenz. Über verschiedene Geschäftsführer-Stationen in der IT-Welt wechselte ich im August 2015 als Bereichsvorstand Technology (CIO) zu OTTO und verantworte seitdem die Weiterentwicklung der IT-Landschaft und des ca. 1000-köpfigen Tech-Teams bei OTTO. Zusätzlich engagiere ich mich ehrenamtlich als Vorstands‑, Präsidiums- oder Beiratsmitglied in unterschiedlichen Tech-Initiativen (The Interface Society, IT Executive Club, Uni Hamburg Stiftungsprofessur IT-Management & Consulting, VOICE, Hamburger IT-Strategietage) und fördere die Vernetzung, den Austausch und Ausbau von IT-Expertinnen, IT-Experten und Tech-Wissen. Meine Herzensangelegenheit: Mehr Frauen in Tech-Berufen. Deshalb setze ich mich aktiv dafür ein, mehr Weiter- und Ausbildungsangebote für Mädchen und Frauen zu etablieren und für Tech-Themen zu begeistern. Außerdem bin ich glücklich verheiratet und stolzer Vater von drei Kindern.

### Rolf Olmesdahl:

Ich war bis 23. November 2021^2^ COO und CIO, Leiter Departement IT & Services und Mitglied der Geschäftsleitung von Raiffeisen Schweiz. Raiffeisen ist die drittgrößte Bankengruppe in der Schweiz und zählt rund 1,95 Mio. Genossenschafterinnen und Genossenschafter sowie 3,6 Mio. Kundinnen und Kunden. Die 219 rechtlich autonomen und genossenschaftlich organisierten Raiffeisenbanken sind in der Raiffeisen Schweiz Genossenschaft zusammengeschlossen. Als CIO bin ich primär für die operative und strategische Leitung der IT sowie die Planung, den reibungslosen Betrieb und die Weiterentwicklung der unternehmenseigenen IT-Architektur und der zentralen IT- und Banking-Systeme verantwortlich.

### Ursula Soritsch-Renier:

Saint-Gobain ist als Unternehmen mit mehr als 167.000 Mitarbeitenden und auf der ganzen Welt aktiv. Ich bin Group Digital and Information Officer im Executive Committee und verantworte die gesamte Digitalisierung und IT der Gruppe. Es gibt neben der IT für die Gruppe noch regionale IT-Organisationen und regionale CIOs, die die jeweiligen lokalen Geschäftsbereiche in ihren spezifischen Bedürfnissen unterstützen.

### Patrick Naef:

Ich bin Managing Partner bei Boyden Global Executive Search. Zudem sitze ich im Verwaltungsrat der Franke Group sowie in einigen kleineren IT-Firmen in der Schweiz und in Deutschland. Ich unterstütze auch Technologie Start-ups und berate Firmen im Bereich der digitalen Transformation. Ich war selbst lange CIO, zuletzt mehr als 12 Jahre bei der Emirates Airline Group in Dubai mit mehr als 100.000 Mitarbeitenden, wo ich über 3000 Mitarbeitende in meiner IT-Division führte.

### *Walter Brenner:*

*Digitalisierung steht heute weit oben auf der Prioritätenliste von fast jedem Unternehmen, unabhängig von der Größe und Branche. Vor diesem Hintergrund stelle ich die Frage: Wie ist der Stand der Digitalisierung in Ihrem Unternehmen Ende 2021 und was sind die größten Erfolge in der Digitalisierung, die Ihr Unternehmen in den letzten Jahren erreicht hat?*

### Hanna Hennig:

Siemens ist es gelungen, einzigartige Kompetenz darin aufzubauen, die digitale und die reale Welt miteinander zu verbinden. Mit dieser Kompetenz kann Siemens seine Kundinnen und Kunden wie kein anderes Unternehmen bei der digitalen Transformation unterstützen. Dazu haben wir unser Hardware-Geschäft mit umfassenden Investitionen in Software und IoT (Internet of Things) weiter vorangetrieben. Auch heute noch investieren wir rund 50 % unserer 4,6 Mrd. € Ausgaben für Forschung und Entwicklung in Software und IoT und setzen alles daran, ein starkes Ökosystem aufzubauen. Besonders stolz sind wir auf die herausragende Position, die wir im Bereich IT/OT-Konvergenz erreicht haben. So sind wir heute führend in der Fabrikautomatisierung mit 45 Mio. installierten Automatisierungssystemen, in der Automatisierung des Schienenverkehrs mit ca. 14.000 Schienenfahrzeugen und der Automatisierung der Netze sowie in der Softwareentwicklung für die Industrie mit ca. 200.000 Entwicklerinnen und Entwicklern bei Mendix, unserer Low-Code-Plattform.

### Andreas Maier:

Die AXA Schweiz hat in den vergangenen Jahren in der Digitalisierung sehr viel erreicht. So haben wir beispielsweise digitale Omni-Channel-Möglichkeiten für alle Leben- und Nichtleben-Produkte geschaffen, für eine komplette digitale Unterstützung des Vertriebs mit digitaler Beratung und Sales für alle Geschäftsbereiche gesorgt und eine Million Leads aus 76 automatisierten datengetriebenen Modellen generiert. Wir haben heute 800.000 Kunden und Kundinnen auf dem myAXA-Portal sowie eine komplett agile Business- und IT-Entwicklung mit 50 Produkte-Teams. Die AXA hat die noimos AG gegründet, ein InsurTech für Computer Vision, Augmented Reality und komplexe Machine-Learning-Lösungen. Ich bin Verwaltungsratspräsident dieses Unternehmens. Stolz bin ich auf die hervorragenden Ergebnisse des Capability Assessment von McKinsey. Wir haben es von „good“ nach „great“ geschafft. Trotz aller dieser Innovationen haben wir eine ausgezeichnete Kostenposition: Wir haben gemäß einem Benchmark mit vier anderen Schweizer Versicherungen 0,3 % tiefere Kosten als der Schweizer Durchschnitt.

### Michael Müller-Wünsch:

OTTO hat es verstanden, den allgemeinen Digitalisierungsschub sowohl in den internen Strukturen, aber auch in der Marktbearbeitung in den vergangenen 24 Monaten maximal zu nutzen. Die schon seit 2016 gestartete Digitalisierung der Employee Journey durch den konsequenten, weitumfassenden Einsatz digitaler Kollaborationswerkzeuge und Workflow-Systeme hat 2021 sicherlich zu einer selbstverständlichen Nutzung digitaler Werkzeuge und Infrastrukturen geführt. Aber nicht nur in den typischen allgemeinen Büro- und Administrationsbereichen, sondern auch in den Technologie-Bereichen haben New Work und Work-From-Home-Methoden und -Konzepte umfassend Eingang gefunden. Selbst agile Entwicklungsmodelle über Kontinente hinweg mit unseren Technologie-Partnern konnten wirksam vorangebracht werden. Die Geschwindigkeit unserer digitalen Transformationsreise mussten wir kaum reduzieren.

Auch in der Marktbearbeitung konnten wir durch die Einführung unseres digitalen Marktplatzes unseren Geschäftsmodellumbau zur hybriden Plattform erfolgreich voranbringen. Als MVP^3^ gestartet, haben wir uns in den vergangenen Monaten sehr erfolgreich neue Erlösströme erschlossen und viele Millionen neue Endkundinnen und Endkunden dazugewonnen. Unsere Digitalisierungsstrategie hat hier ebenso erfolgreich Wirkung erzielt. Und mit unserer Initiative „Pushing AI“ haben wir einen Leuchtturm in der teilautomatisierten, datengetriebenen Unternehmenssteuerung definieren und umsetzen können.

### Rolf Olmesdahl:

Raiffeisen hat in den letzten Jahren markante Fortschritte bei der „Digitalen Durchdringung“ erzielt. Die Anzahl von Transaktionen, die durch Kundinnen und Kunden im Rahmen eines vollautomatisierten Prozesses abgewickelt wird, ist stark gestiegen. Immer mehr Produkte und Dienstleistungen werden auch auf den digitalen Kanälen angeboten. Die Frequenz auf dem physischen Verkaufskanal hat sich entsprechend reduziert. Im Rahmen der Strategie „Raiffeisen 2025“ investiert Raiffeisen substanziell in den weiteren Ausbau ihrer digitalen Kanäle. Aktuell arbeitet Raiffeisen an der Entwicklung eines Kundenerlebnis-Portals, in dem alle digitalen Dienstleistungen im Sinne eines Self-Service gebündelt werden. Für die Mitarbeitenden hat Raiffeisen einen digitalen Workplace geschaffen, der das vollständig ortsunabhängige Arbeiten ermöglicht. Beschleunigt durch die Pandemie hat sich damit auch die Kultur und die Arbeitserledigung in Richtung von hybriden und agilen Methoden verändert.

### Ursula Soritsch-Renier:

Die Bauindustrie hat als einzige Industrie fast weltweit in den letzten fünfzig Jahren an Produktivität verloren. Kaum zu glauben, es ist aber so. Digitalisierung ist ein absolutes Muss und ein wichtiges Instrument, um Fortschritte bei der Produktivität zu erzielen. Saint-Gobain gehört nach dem Clarivate Top 100 Global Innovator Rating seit zehn Jahren zu den hundert innovativsten Firmen der Welt und ist daher in ausgezeichneter Position, diese Herausforderungen zu meistern. Das Unternehmen hat einen hochtechnischen Spezial-Mörtel für 3D-Drucker entwickelt, der bei dem ersten Haus, das vollständig aus 3D-gedrucktem Beton hergestellt wurde, zum Einsatz kam. Dieses Haus steht in Holland, Menschen, die man besuchen kann, leben darin. Wir sind sehr innovativ im Bereich Building Information Models, Echtzeitkommunikation und Product Information Systeme. Wir haben Software entwickelt, die wir unseren Kundinnen und Kunden offerieren, um ihnen bei Ausschreibungen zu helfen. Wir verwenden in Kanada Sensoren im Beton, die überwachen, wie der Beton trocknet. Durch diese Technologie kann bis zu 90 % der Nacharbeit nach dem Betonieren vermieden werden, um einige digitale Innovationen zu nennen. Ich denke aber, dass es eine Herausforderung bei Saint-Gobain ist, wie für viele andere Unternehmen, diese Innovationen innerhalb des Konzerns zu verbreiten und zu skalieren.

### Patrick Naef:

Viele CIOs lassen sich feiern, dass sie innert kürzester Zeit die nötige Infrastruktur für Homeoffice bereitstellen konnten. Jedoch einfach MS-Teams/M365 einzuführen und ein paar Server in die Cloud zu verschieben hat nicht wirklich einen transformativen Charakter für das Geschäft der jeweiligen Firmen. Bzgl. neuer Geschäftsmodelle, Distributionskanäle, Digitalisierung und Personalisierung der Produkte etc. hat sich bei den meisten Firmen sehr wenig getan und der Großteil der CIOs scheint sich noch immer (oder wieder vermehrt) auf nicht-strategische Themen wie Infrastruktur, Cyber Security und ERP-Upgrades zu konzentrieren. So wurde auch im November 2021 treffend in der Zeitschrift Bilanz aus einer Studie eines Beratungsunternehmens berichtet: „Mehr als 80 % der IT-Investitionen fließen in Digitalisierungsprojekte ohne transformativen Charakter.“

### *Walter Brenner:*

*Ihre Antworten zeigen klar, dass Digitalisierung in Ihren Unternehmen angekommen ist und Digitalisierung mehr ist als die klassische Automatisierung von Prozessen durch den Einsatz von ERP-Software *(Leimeister et al. 2014)*. Die Unternehmen, die von Ihnen vertreten werden, entwickeln neue digitale Dienstleistungen, virtualisieren Produkte und sind auch dabei, ihre bestehenden Geschäftsmodelle zu hinterfragen und neue zu entwickeln. In unserer Forschung sehen wir, dass viele CIOs nicht nur, wie in der Vergangenheit, bei Prozessautomatisierung, sondern auch bei der Entwicklung neuer digitaler Dienstleistungen, bei der Virtualisierung von Produkten und auch bei der Schaffung neuer Geschäftsmodelle mitwirken wollen. Gleichzeitig sehen wir in unseren Erhebungen, dass ein stabiler Betrieb, das Sicherstellen geringer Antwortzeiten, das erfolgreiche Durchführen großer Projekte und die Gewährleistung von IT-Sicherheit viele CIOs stark beschäftigt. Vor diesem Hintergrund meine Frage: Welche Rolle spielen Sie als CIO bei der Digitalisierung Ihres Unternehmens?*

### Hanna Hennig:

Erste Priorität eines CIO muss es sein, die Systemlandschaft seines Unternehmens stabil und sicher bereitzustellen, damit es produktiv bleibt. Insbesondere in Krisenzeiten, wie zuletzt während der COVID-19-Pandemie, hat sich gezeigt, dass die IT das Rückgrat eines Unternehmens ist. Dennoch muss ich als Unternehmerin – so verstehe ich mich als CIO und führe „IT as a Business“ – sicherstellen, dass wir in der IT genügend Ressourcen und Zeit investieren, um die Unternehmensstrategie von Siemens zu unterstützen und insbesondere mit Digitalisierungsmaßnahmen wettbewerbsfähig und erfolgreich zu gestalten. Zusammen mit den Fachbereichen erstellen wir Roadmaps für die Digitalisierung unserer Geschäftsprozesse und Werke. Wir entwickeln und ermöglichen neue digitale Produkte und Geschäftsmodelle. Zudem machen wir IT anfassbar und einfach nutzbar, damit jede Mitarbeiterin und jeder Mitarbeiter befähigt ist, bei der digitalen Transformation aktiv mitzuwirken. Denn die Digitalisierung eines Konzerns ist ein unternehmensweites Vorhaben und kann nicht allein mithilfe einer Einheit gelingen. Dazu gehören Hilfsmittel wie Low Code und im Rahmen unserer Digital-Citizen-Strategie Citizen-Developer-Konzepte sowie personalisierte Trainings zu digitalen Technologien für die jeweiligen Jobprofile, beispielsweise in Vertrieb, Controlling, HR und Produktion.

### Andreas Maier:

Da ich in der AXA Schweiz verantwortlich bin für IT, für Data Driven Company und Innovationen bin ich die zentrale Person in der Geschäftsleitung, die Digitalisierung treibt.

### Michael Müller-Wünsch:

Als gleichberechtigtes Mitglied des Bereichsvorstands der Marke OTTO verantworte ich gemeinschaftlich mit meinen Kollegen und Kolleginnen den Unternehmenserfolg und die Zukunftsgestaltung unseres Unternehmens. Bei OTTO findet die Digitalisierung in allen Bereichen des Unternehmens tagtäglich statt. Insofern gibt es keinen Wettbewerb um den Digitalisierungshut. Jede Bereichsvorständin und jeder Bereichsvorstand verantwortet in seiner oder ihrer Bereichsstruktur die Kerndisziplin und trägt damit zum Gesamterfolg des Unternehmens bei. Als Bereichsvorstand Technology gehört selbstverständlich der reibungslose und effiziente Betrieb der Technologielandschaften dazu, genauso aber auch die Umsetzung der Change- und Innovationsinitiativen in allen Bereichen.

### Rolf Olmesdahl:

Digitalisierung ist Aufgabe der ganzen Geschäftsleitung und des ganzen Teams. Der CIO nimmt dabei eine wesentliche Rolle ein, da er in der Regel innerhalb der Geschäftsleitung am nähesten an der Thematik dran ist. Mit der weiter fortschreitenden Digitalisierung ist davon auszugehen, dass auch die businessorientierten Geschäftsleitungsmitglieder dem Thema vermehrt Bedeutung beimessen und entsprechend öfter als Auftraggeber gegenüber der IT auftreten, wie dies bei der Raiffeisen der Fall ist.

### Ursula Soritsch-Renier:

Da ich im Executive Committee sitze, bin ich die oberste strategische Instanz für Digitalisierung. Mit Digitalisierung müssen sich alle Mitarbeitenden im Unternehmen beschäftigen. Ich bin sowohl Chief Information als auch Chief Digital Officer, da sich, meiner Meinung nach, diese beiden Rollen nicht voneinander trennen lassen. Es geht konkret um vier Aufgaben: Die erste Aufgabe ist der Aufbau von Grundlagen für die Digitalisierung. Das bedeutet beispielsweise, an der Datenintegrationsarchitektur zu arbeiten oder Community-Plattformen einzurichten, damit sichtbar wird, wo welche Innovationen stattfinden. Die zweite Aufgabe ist der Aufbau von „Deep Expertise“. Ich werde eine digitale Forschung und Entwicklung aufbauen, ähnlich wie beispielsweise Pharma eine Forschung und Entwicklung für pharmazeutische Produkte besitzt. Ein Unternehmen wie Saint-Gobain braucht Deep Expertise beispielweise für Blockchain, Augmented Reality, Virtual Reality und mehr. Aufgabe drei ist der Aufbau von Plattformen, die auf der einen Seite weltweit eingesetzt werden können, auf der anderen Seite aber funktional an die lokalen Bedürfnisse, beispielsweise von Brasilien, Frankreich, der Türkei oder der USA, angepasst werden können. Die Märkte sind sehr unterschiedlich. Das bedeutet, dass die Zentrale die Verantwortung für die Plattform hat, die „last Mile to the Customer“ ist die Entscheidung der lokalen Organisationen. Wenn die Zentrale für die ganze Gruppe zum Beispiel eine Augmented-Reality-Plattform aufbaut, kann diese im Training für Kunden oder für das Facility Management in Fabriken oder für den Verkauf verwendet werden. Kunden können in der virtuellen Welt sehen, wie ein Gebäude später aussehen wird. Schlussendlich geht es bei Aufgabe vier um die agile Entwicklung und den Betrieb von Applikationen, wo wir mit „cloud first“ eine neue Richtung für Saint-Gobain einschlagen.

### *Walter Brenner:*

*Patrick Naef, Sie sind hauptamtlich mit der Suche nach Führungskräften und CIOs beschäftigt. Welche Anforderungen an CIOs stellen Ihre Kunden?*

### Patrick Naef:

Viele Unternehmen sehen, dass ihr CIO sich primär auf die Disziplin der Prozess-IT konzentriert und andere Aufgaben zu kurz kommen. Das reicht heute nicht mehr aus. Die kurze Phase, während derer ein CDO (Chief Digital Officer) angestellt wurde, um die Produkt-Digitalisierung voranzutreiben ist endgültig vorbei. Niemand stellt heute noch einen CDO an, im Gegenteil, die Rollen CIO und CDO werden kombiniert. Auch wenn es gewisse Unternehmen nicht genauso formulieren können, beobachte ich doch überall dasselbe Muster: Man will die Disziplinen kombinieren und sucht eine Führungskraft, die dem gewachsen ist, eine Person, die es versteht, eng mit den Kolleginnen und Kollegen in den Geschäftsbereichen zusammenzuarbeiten, d. h. sehr geschäftsorientiert, strategisch und unternehmerisch, unhierarchisch und in vernetzten Strukturen denken kann und das volle Potenzial der Technologie für alle Aspekte des Unternehmens versteht. Diese Suchen werden meist vom CEO und/oder sogar vom Verwaltungsrat getrieben.

### *Walter Brenner:*

*Die Antworten auf die Frage nach der Verantwortung des CIO im Rahmen der Digitalisierung ergeben ein klares Bild. Der CIO trägt große Verantwortung bei der Digitalisierung. Auf der einen Seite geht es um die Bereitstellung der notwendigen Infrastrukturen sowie deren Entwicklung und Betrieb und auf der anderen Seite um die Mitarbeit an digitalen Innovationen und die Gestaltung der Zukunft von Unternehmen. Bereits haben führende CIOs den „Keller“ verlassen und sind zu einem Partner der Fachbereiche geworden, um Potenziale der Informations- und Kommunikationstechnik frühzeitig zu erkennen und zu realisieren*.

*Es gibt jahrzehntelange Diskussionen über den CIO, seine Aufgaben und seine Zukunft. Generationen von CIOs, Beraterinnen und Berater sowie Wissenschaftlerinnen und Wissenschaftler, zu denen ich auch gehöre, haben sich mit dem CIO beschäftigt. Der CIO wurde schon mehrfach „totgesagt“. Aber wie so oft: „Totgesagte leben länger.“ Auch wenn Sie diese Frage vielleicht nicht mehr hören können, gibt es 2030 noch CIOs?*

### Hanna Hennig:

Ja, es wird weiterhin den oder die CIO geben, denn die IT ist heute wichtigster Technologie-Enabler und zugleich Impulsgeber, Visionär und Business-Partner globaler Konzerne. Technologie und IT sind bereits jetzt schon wesentliche Teile der Strategie und des Geschäfts. Um dieser bedeutenden Rolle der IT gerecht zu werden, braucht es mehr denn je eine derartige Schlüsselposition, die Technologiekompetenz und unternehmerisches Know-how in einer Funktion abbildet. Allerdings werden sich die Aufgaben von CIOs in den nächsten Jahren verändern: Bislang waren sie vorwiegend damit beschäftigt, Technologien für die Arbeitsplatzumgebung, die Office-IT, bereitzustellen und Geschäftsprozesse mit der Prozess-IT zu automatisieren. Vorwiegend werden dafür standardisierte Technologie-Pakete wie ERP-Systeme verwendet. Mit den neuen digitalen Technologien wie Cloud, Data Analytics und KI eröffnen sich nun gänzlich neue Tätigkeitsfelder: In der Fertigung konvergieren IT und OT. CIOs sind damit viel stärker in die Produktions-IT eingebunden. Anderseits entwickeln sie gemeinsam mit den Business-Einheiten neue Produkte und sogar neue Geschäftsmodelle. Das heißt, CIOs sind nun auch in der Produkt-IT und in der Business-Model-IT tätig.

### Andreas Maier:

Ja, es wird auch 2030 noch CIOs geben. Sie werden zusätzlich das Datenthema, mehr Innovation und mehr Business-Modelle verantworten sowie mehr Transformationsarbeit leisten. Technology und Data müssen zusammengeführt werden, denn nur gemeinsam schaffen sie mehr Unternehmenswert.

### Michael Müller-Wünsch:

Früher wurde Technologie – damals noch IT – als ein rasant wachsender Kostenfaktor in den Organisationen gesehen. Geschäftsführungen sahen hier ihre Hauptaufgabe darin, diesen Ressourcenverbrauch bestmöglich zu steuern bzw. zu reduzieren. Über Jahre hinweg befanden sich viele IT-Verantwortliche in der Rechtfertigungsdiskussion zu den IT-Ausgaben – immer gepaart mit dem großen Wunsch an die IT-Organisation, sich doch stärker an den Business-Fragestellungen zu orientieren. Dies hat sich spätestens mit dem Jahr 2020 verändert. Es ist deutlich zu erkennen, dass ein gleichberechtigterer Dialog zwischen den Business- und den Tech-Communities stattfindet. CIOs werden viel häufiger in den gesamtunternehmerischen Gestaltungsauftrag der Unternehmen eingebunden. Mancherorts wird vom Verschmelzen und Integrieren von Technologie und Business im Kontext von agilen, produktorientierten Organisationen gesprochen. Diesen Prozess zu moderieren, mit Augenmaß und Vernunft voranzutreiben, wird mehr und mehr zur Aufgabe moderner CIOs, die weiterhin über eine hohe technologische Kompetenz verfügen müssen und sollten.

### Rolf Olmesdahl:

Ja, auch 2030 wird es die CIO-Rolle noch geben. Die Digitalisierung wird noch mehr technische Lösungen hervorbringen. Diese müssen, auch wenn sie auf Cloud- oder alternativen Plattformen basieren, professionell gemanagt werden. Die Aufgabe des CIO wird sich jedoch verändern. Der Anteil der Eigenentwicklungen wird sich reduzieren, während das Provider- und Service-Management an Bedeutung gewinnen wird.

### Ursula Soritsch-Renier:

Ich bin zu 100 % der Meinung, dass es 2030 noch eine oder einen CIO geben wird. IT wird immer zu einem strategischen Element des Geschäfts gehören. Vor zehn Jahren haben wir E‑Commerce gesagt, und heute sagen wir digital. Die Bedeutung der Informations- und Kommunikationstechnik wird weiter steigen. Dass ich im Executive Committee sitze, beweist die Bedeutung der Digitalisierung für Saint-Gobain.

### Patrick Naef:

Es geht meines Erachtens weniger um den Titel oder Begriff, sondern mehr um eine Rolle im Unternehmen. Auch wenn man heute schon erwartet, dass jede Führungskraft sich um ihre personellen und finanziellen Ressourcen kümmert, obwohl es einen Chief Financial Officer und Chief Human Resources Officer gibt, muss sich auch jede Führungskraft vermehrt um Technologie als strategische Komponente kümmern, obwohl es noch eine oder einen CIO (oder ähnlich) gibt, der oder die die Klammer über alle Technologie-Themen im Unternehmen bildet. Klar ist aber, dass sich die strategische Bedeutung der IT massiv verstärkt hat und sich somit die Rolle des CIO vom traditionellen IT-Chef, der oder die sich im Backoffice um Server, PCs, Netzwerke etc. kümmerte, hin zu einer strategischen Rolle im Kern des Geschäftes entwickeln muss. Ich bin überzeugt, dass die Rolle des CIO noch nie so entscheidend war wie heute.

### *Walter Brenner:*

*Die CIOs, die an diesem Dialog beteiligt sind, sind sich einig: Es wird auch im Jahr 2030 noch einen CIO geben. Diese Antworten spiegeln auch das Selbstbewusstsein wider, das die CIOs durch die gute Arbeit in der Pandemie gewannen. Selbstzweifel an ihrer Rolle und an ihrer zukünftigen Bedeutung existieren im Moment nicht *(Brenner et al. 2021)*. Die CIOs sind sich sicher, dass sie die Gewinner der Digitalisierung sein werden.*

*Gehen wir noch einen Schritt weiter im Dialog. Seit einigen Jahren gibt es in vielen Unternehmen eine Position, die sich um Digitalisierung kümmert, den Chief Digital Officer (CDO). Gibt es in Ihrem Unternehmen einen CDO? Wenn ja, wie gestaltet sich die Zusammenarbeit mit dem CDO?*

### Hanna Hennig:

Den Titel CDO hat bei Siemens bisher niemand für sich beansprucht. Für mich sind die Funktionen, die der CDO in anderen Unternehmen übernimmt, bei Siemens Teil meiner Rolle als CIO.

### Andreas Maier:

Bei der AXA Schweiz gibt es keinen CDO.

### Michael Müller-Wünsch:

Bei der Marke OTTO wurde keine Rolle als CDO etabliert. Meine persönliche Einschätzung hierzu ist, dass bei einem kollegialen, respektvollen Umgang der Digital-Verantwortlichen und derer Bereiche ein CIO/CDO-Duo nur zur notwendigen Stärkung der Digitalkompetenz eines Unternehmens beitragen. Wenn diese Wertschätzung nicht existiert, ist es aus meiner Perspektive eine vergebliche Investition.

### Rolf Olmesdahl:

Bei Raiffeisen gibt es keinen CDO. Raiffeisen ist der Auffassung, dass Digitalisierung Aufgabe der ganzen Geschäftsleitung ist.

### Ursula Soritsch-Renier:

Ich bin in Personalunion CIO und CDO.

### *Walter Brenner:*

*Patrick Naef, gibt es aus Ihrer Sicht einen Trend, eher CDOs als CIOs zu suchen? Was erleben Sie in den Gesprächen, wenn es um die Ausgestaltung von Jobs für CIOs und der Festlegung der Suchprofile geht?*

### Patrick Naef:

Wie ich oben erwähnte, sucht heute niemand mehr einen reinen CDO, diese Positionen werden überall wieder abgebaut. Vielmehr suchen Firmen Personen, die alle Disziplinen der IT oder der digitalen Technologien verstehen und zusammenbringen können. Zudem muss jede Führungskraft zu einem „Digital Leader“ werden.

### *Walter Brenner:*

*Die Antworten auf die Frage nach dem CDO und seinem Verhältnis zum CIO verstärken die Antworten auf die letzte Frage. In den Unternehmen, deren CIOs an diesem Dialog mitmachen, gibt es keinen CDO und es ist auch nicht geplant, eine solche Stelle zu schaffen. Die Verantwortung für die Digitalisierung ist und bleibt beim CIO.*

*CIOs und Beratungsunternehmen, auch die Gartner Group, berichten übereinstimmend, dass immer mehr Verantwortung und Budget für IT in die Fachbereiche verschoben werden (Panetta* 2021*). Sehen Sie diesen Trend in Ihrem Unternehmen? Wie beurteilen Sie diese Verschiebung, einfach wieder mal ein Aufleben der Diskussionen um Schatteninformatik oder eine spannende wichtige Entwicklung? Ist dies für Sie eher Notwendigkeit, Chance oder Gefahr?*

### Hanna Hennig:

In meinen Augen ist dieser Trend genauso volatil wie der Trend zur Zentralisierung bzw. Dezentralisierung von IT. Ich halte nichts davon IT-Verantwortung und Budget in die Fachbereiche zu verschieben, da die Kernkompetenz zum sicheren und stabilen Betrieb nun mal bei der IT liegt. Ebenso sollte die Buchhaltung nicht in die IT ausgelagert werden, weil es nicht Kernkompetenz der IT ist, am Monats- oder Jahresende die Bücher zu schließen. Was ich jedoch sehr wohl unterstütze und mit meinem Team fördere, ist die „Demokratisierung“ der IT. Wir statten Mitarbeitende mit digitaler Kompetenz aus, damit sie die verfügbare Technologie besser für ihre Arbeit nutzen können, beispielsweise stellen wir für die Datenanalyse einen „Data Lake to Go“ bereit. Ebenso unterstützen wir die Entwicklung von „Citizen Developern“, technikaffinen Mitarbeitenden, die mithilfe unserer Low-Code-Plattform Mendix Software entwickeln können, ohne dass sie dafür ein Informatikstudium absolvieren mussten. Damit erweitern wir die Anzahl der Mitarbeitenden, die die digitale Transformation im Unternehmen vorantreiben.

### Andras Maier:

Nein, diesen Trend kann ich nicht bestätigen. In der AXA werden Investitionen gesamthaft betrachtet. Die Entscheidung und wieviel investiert wird, findet zentral mit der Geschäftsleitung statt. Die AXA investiert in Zeiten der Digitalisierung im Vergleich zu anderen Versicherern in der Schweiz ca. 5 % mehr in Change, trotz 0,3 % tieferer GWP/IT Cost Ratio. Investitionen in die sog. Schatten-IT finden wenig statt, da sie außerhalb der agilen Governance laufen und automatisch Probleme generieren würden.

### Michael Müller-Wünsch:

Diese Entwicklung ist bei OTTO nicht zu beobachten. Technologie wird bei uns architektonisch ganzheitlich betrachtet, gestaltet und gesteuert. Hierzu haben wir einen unternehmensweit gültigen Portfolio- und Priorisierungsprozess definiert, der vom Bereichsvorstand gemeinsam gesteuert und verantwortet wird. Budgets werden entsprechend der Unternehmenszielstellungen allokiert. Mit einer hinreichenden, gesamt-unternehmerischen Transparenz soll so möglichen Fehlentwicklungen entgegengewirkt werden.

### Rolf Olmesdahl:

Diesen Trend kann ich nicht bestätigen. Größere Projekte werden bei Raiffeisen, auch im Zeitalter der Digitalisierung, noch immer in einem Joint-Venture zwischen Business und IT umgesetzt.

### Ursula Soritsch-Renier:

Saint-Gobain hat eine sehr dezentrale Organisation. Die regionalen Organisationen haben teilweise große Budgets. Ist das gut oder ist das schlecht? Ich glaube, das derzeitige Setup ist für einen globalen Konzern wie Saint-Gobain eine gute Organisation. Zentralisierung wie auch Dezentralisierung haben Vor- und Nachteile. Am Ende geht es darum, wie man miteinander arbeitet und miteinander umgeht. Ich habe in meiner bisherigen Karriere in den unterschiedlichsten Setups gearbeitet – zentralisiertes oder dezentralisiertes Budget. Bei Saint-Gobain arbeite ich in einer mehrheitlich dezentralen Geschäftsstruktur, wobei ich als Gruppenfunktion eine wesentliche Aufgabe bei der Standardisierung und Optimierung und als Enabler für digitale Innovation habe. Beides ist möglich. Es kommt auf die Unternehmenskultur an und wie die Governance-Struktur aufgesetzt ist. Ich habe in meiner beruflichen Laufbahn noch nie gesehen, dass ein Unternehmen kein Geld für etwas wirklich Gutes hat. Wenn es das Richtige für das Unternehmen ist, sollte das „Dies gehört mir“ und „Dies gehört nicht dir“ keine Rolle spielen. Das Wichtigste ist, das Richtige zu machen. Es geht bei Saint-Gobain darum „Let’s find the best solution for the company“.

### *Walter Brenner:*

*Patrick Naef, wie sehen Sie die Verschiebung von Verantwortung und Budget für IT in die Fachbereiche? Spielt dies eine Rolle bei der Suche nach CIOs?*

### Patrick Naef:

Noch vor zehn Jahren hatte ich in meiner Funktion als CIO auch gegen Schatten-IT gekämpft. Die weit verbreitete Ansicht war, dass alles was die IT betrifft, zentral vom CIO und seiner IT-Organisation kontrolliert und gesteuert werden müsse. Begründet wurde dies durch Security- und Risiko-Aspekte, Kostenoptimierung, Vermeidung von Doppelspurigkeit, Standardisierung etc. Zwar konnten die Kosten durch die Zentralisierung der gesamten IT optimiert werden, die Kehrseite war jedoch, dass Geschwindigkeit, Innovationskraft und Nähe zum Geschäft und den Kunden unter einem solchen Ansatz litten. Schatten-IT-Teams hingegen sind in der Regel viel näher am Geschäft – sie sind sogar vollständig ins Business integriert – und verstehen das Geschäft und die Kunden besser als zentrale IT-Organisationen. Zudem sind sie aufgrund ihrer geringeren Größe und ihres begrenzten Overheads typischerweise viel schneller und agiler, um auf Marktgegebenheiten zu reagieren.

Vielleicht sollten Schatten-IT-Teams von CIOs eher als Segen, denn als Fluch angesehen werden, solange die CIOs offen für eine Zusammenarbeit mit ihnen sind. Die Tatsache, dass Führungskräfte im Business Schatten-IT-Teams unterstützen und verteidigen, zeigt, dass sie sich um die IT kümmern und sie diese als eine wesentliche und strategische Komponente ihres Geschäfts betrachten und somit genau auf dem richtigen Weg sind. CIOs müssen in der Lage sein mit Schatten-IT-Teams im gesamten Unternehmen zusammenzuarbeiten, von deren Nähe und Verständnis für das Geschäft sowie von deren Flexibilität und Innovationskraft zu profitieren, ohne dabei Sicherheit zu gefährden oder gar in ein unkontrolliertes Chaos abzudriften.

### *Walter Brenner:*

*Die Antworten nach der Verlagerung von Verantwortung und Budget für IT in die Fachbereiche wird unterschiedlich beantwortet. Dieses uneinheitliche Bild steht im Widerspruch zu vielen Aussagen von Beratungsunternehmen und der Wissenschaft, die von einer irreversiblen Verschiebung von Verantwortung und Budget in die Fachbereiche ausgehen (Panetta* 2021*). Zentralisierung und die mit ihr leichtere Standardisierung scheint nach wie vor Vorteile zu haben und zentrale Entscheide scheinen geeignet, eine optimale Allokation der Investitionen zu ermöglichen. Den in einigen Unternehmen feststellbaren Trend zu mehr Verantwortung und Budget für IT in den Fachbereichen gilt es in den nächsten Jahren zu beobachten, um herauszufinden, ob es sich nur um eine Modeerscheinung handelt oder ob hier tatsächlich ein wichtiger Transformationsprozess im Gange ist. Interessant ist aus meiner Sicht, dass sich das Verhältnis zur Schatten-IT entspannt hat und sie von den CIOs als einer der Motoren für Innovation gesehen wird.*

*Wie gestaltet sich Ihre Zusammenarbeit mit dem CEO bzw. der Geschäftsleitung? Sind Sie Teil der Geschäftsleitung? Wenn ja, wie bringen Sie Ihre Themen in die Sitzungen der Geschäftsleitung ein? Wenn nein, haben Sie genügend Zugang zu den hierarchisch höchsten Gremien Ihres Unternehmens?*

### Hanna Hennig:

Als CIO berichte ich direkt an den Vorstand und habe uneingeschränkten Zugang. In meiner Rolle bin ich Teil der strategischen IoT-Produktentwicklungen und weiterer Gremien, in denen die Digitalisierung des Konzerns vorangetrieben wird.

### Andreas Maier:

Ich bin seit 9,5 Jahren in der Geschäftsleitung. Die Zusammenarbeit in der Geschäftsleitung ist seit sehr vielen Jahren auf Augenhöhe. Als CIO bringe ich TechnologieThemen, technologiegetriebene Business Cases, die IT-Strategie, Informationssicherheit, Möglichkeiten der Steigerung der Effizienz und Strategien für ein datengengetriebenes Unternehmen in die Geschäftsleitung ein.

### Michael Müller-Wünsch:

Als gleichberechtigtes Mitglied des Bereichsvorstands der Marke OTTO habe ich keine Probleme, die Technologie-Themen in die Vorstandssitzungen einzubringen. Die Zusammenarbeit mit meinen Vorstandskolleginnen und -kollegen gestaltet sich hinreichend problemlos, ohne dass diese Personen über einen vergleichbaren Technologie-Background wie der Ressortverantwortliche verfügen. Hier wird auf meine Fachkompetenz als Informatiker und meine Gesamtverantwortung für das Gesamtunternehmen seit Jahren vertraut.

### Rolf Olmesdahl:

Der CIO ist bei Raiffeisen Schweiz Mitglied der Geschäftsleitung. Er hat damit genau den gleichen Zugang zum CEO und dem Verwaltungsrat wie die anderen Geschäftsleitungsmitglieder.

### Ursula Soritsch-Renier:

Wie bereits erwähnt, bin ich Mitglied des Executive Committee. Wenn ich etwas auf einer Geschäftsleitungssitzung besprechen möchte, bekomme ich die entsprechende Zeit.

### *Walter Brenner:*

*Patrick Naef, Sie haben täglich mit CEOs und Verwaltungsratspräsidenten zu tun. Wie sehen diese Persönlichkeiten ihre CIOs und wie gehen diese Führungskräfte mit Digitalisierung um?*

### Patrick Naef:

Jede und jeder CEO, der die strategische Bedeutung der IT versteht, will einen CIO, der sehr eng mit dem CEO und der Geschäftsleitung zusammenarbeitet und auch im Verwaltungsrat entsprechende Visibilität hat. Das bedeutet nicht zwangsläufig, dass der CIO auch direkt an den CEO berichtet und in der Geschäftsleitung sitzen muss. Viele Unternehmen werten die CIO-Rolle zwar auf, indem sie diese in die Geschäftsleitung nehmen. Wenn man sich aber vom traditionellen hierarchischen Denken löst, dann erkennt man rasch, dass dies nicht ein notwendiges Kriterium ist, um als CIO erfolgreich zu sein. Ich hatte beispielsweise während den 12 Jahren als CIO bei Emirates auch nicht an den CEO berichtet, war offiziell auch kein Konzernleitungsmitglied; trotzdem war ich bei jeder Konzernleitungssitzung dabei und hatte direkten Zugang und Akzeptanz zu jeder relevanten Führungskraft im Unternehmen. Ich hatte mir meine Netzwerk-Strukturen selbst unabhängig von den Hierarchien aufgebaut.

### *Walter Brenner:*

*Die alte Frage nach dem Zugang des CIO zur Geschäftsleitung wird von den CIOs dieser Runde sehr entspannt gesehen. Entweder sind sie – teilweise schon seit vielen Jahren – Teil der Geschäftsleitung oder sie haben einen direkten Kontakt zum CEO und zur Geschäftsleitung. Die zunehmende Bedeutung der Digitalisierung lässt auch keine andere Antwort auf diese uralte Frage zu.*

*Welche Eigenschaften bzw. welche Kompetenzen sind für Sie in Ihrer Rolle als CIO in Zukunft besonders wichtig? Was sollten die Studierenden, die den Job des CIO anstreben, lernen?*

### Hanna Hennig:

Heute und in Zukunft geht es um viel mehr als nur Technologie. Ein CIO sollte unternehmerisches Know-how, Marktverständnis und technologische Kompetenz mitbringen. Er hat sowohl eine strategische als auch eine operative Rolle. Es geht um Leadership, Intercultural Diversity, Transformation, Change Management sowie um „Run, Change and Engineer the Business“. Um dem insgesamt gerecht zu werden, ist ein hohes Maß an Technologie- und Geschäftsexpertise erforderlich.

### Andreas Maier:

Ein CIO der Zukunft braucht vor allem Verhandlungs- und LeadershipKompetenz. Daneben braucht es Kompetenz in Technologiemanagement, Entwicklung digitaler Geschäftsmodelle sowie in Daten- und Innovationsmanagement.

### Michael Müller-Wünsch:

Neben einer tiefen technologischen Kompetenz und einem guten Technologie-Architektur-Wissen sind Organisationsgestaltungs- und Arbeitsmethodenwissen von großer Hilfe in der erfolgreichen Ausübung einer CIO-Position. Hilfreich ist es für CIOs, wenn sie über Kenntnisse des Geschäftsmodells, für welches sie wirken sollen und wollen, verfügen. Das schafft die Nähe, um als gleichbedeutende Gesprächspartnerinnen und Gesprächspartner sowie Unternehmenslenkerinnen und -lenker wahrgenommen zu werden. Die Rolle des CIO verlangt auch noch Mut, weil viele Situationen unter Unsicherheit bearbeitet werden müssen. Da helfen sowohl profunde Erfahrungen im aktiven Risiko-Management als auch der eine oder andere Fehler, aus dem man hoffentlich gelernt hat.

### Rolf Olmesdahl:

Die Bedeutung von technischen Kompetenzen und dem Projekt-management haben abgenommen. In den Vordergrund gerückt sind Innovations-management, betriebswirtschaftliches Wissen sowie das Orchestrieren von Providern und das Service-Management.

### Ursula Soritsch-Renier:

Grundlage sind für mich zunächst einmal technische Kompetenzen als Basis. Man muss wissen, worüber man spricht, da man ansonsten die Komplexität nicht versteht. Kompetenzen in Projektmanagement und Betrieb sind ein weiterer Bestandteil sowie Personalführung, da es um das Gewinnen und Halten von guten Mitarbeitenden geht. Des Weiteren wird manchmal die Fähigkeit unterschätzt, nach innen und nach außen professionell und gut zu kommunizieren. Nur so wird diese Funktion ein Teil des Geschäfts und keine „Supportfunktion“.

### Patrick Naef:

Die Rolle des CIO wird sich grundlegend ändern. Und zwar weg von der zentralen Kontrolle der IT als operative Support-Einheit hin zu einem Coach, Katalysator und Netzwerker, der es dem Unternehmen ermöglicht, sich in ein digitales Unternehmen zu transformieren. Der CIO muss sich auch aktiv vom traditionellen hierarchischen Denken lösen und ein Vorbild für eine vernetzte Organisation werden. Nicht die Größe der IT-Organisation, gemessen an der Anzahl Mitarbeitenden, oder die Größe des IT-Budgets definiert die Bedeutung der IT und damit die des CIO, sondern der Mehrwert, den der CIO und die IT bringen, und der Impact, den er oder sie auf die Stakeholder des Unternehmens, d. h. das Business, die Mitarbeitenden, Kunden, Umwelt etc., ausüben kann.

### *Walter Brenner:*

*Wenn es um die Kompetenzen geht, die für einen erfolgreichen CIO notwendig sind, führt kein Weg an ausreichender technischer Kompetenz vorbei. Ohne tiefe Kenntnisse der Informatik sind fundierte Entscheidungen nicht möglich. Neben den technischen Kompetenzen spielen menschzentrierte Kompetenzen, wie beispielsweise Leadership, eine große Rolle. In dieser Richtung entspricht das zukünftige Kompetenzprofil dem anderer Topmanager.*

*Zumindest die Studierenden an der Universität St. Gallen fragen immer häufiger, was die Universität und auch ich als Professor mit gesellschaftlicher Relevanz machen. Bei den Fragen spielt Nachhaltigkeit eine große Rolle, aber auch Diversität und Genderfragen werden vermehrt gestellt. Vor diesem Hintergrund meine Frage: Wie stark spielen ökologische Fragen, beispielweise bei Entscheidungen über die zukünftige IT-Infrastruktur, eine Rolle und wie gehen Sie mit Diversifizierung Ihrer Mitarbeitenden um, beispielsweise beteiligen Sie sich an Initiativen zu mehr Frauen in der IT?*

### Hanna Hennig:

IT und Digitalisierung sind Wegbereiter für mehr Nachhaltigkeit, müssen aber auch zwingend unter ethisch korrekten Aspekten und mit Bedacht umgesetzt werden. Wichtige Stichworte sind der massiv steigende Energiebedarf, eine Ethik der Datennutzung und der Umgang mit Elektroschrott.

Bei Siemens ist DEGREE (steht für: Decarbonization, Ethics, Governance, Resource Efficiency, Equity und Employability) als incentiviertes Rahmenwerk gesetzt. Es definiert neben nachhaltigkeitsrelevanten KPIs wie „Decarbonization“ und „Resource Efficiency“ auch die Zielsetzung bei Diversität, Genderfragen und weiteren gesellschaftlichen Themen, die in Summe einen 360-Grad-Blick auf das Thema Nachhaltigkeit ergeben. Green IT, d. h. die Nachhaltigkeit unserer eigenen IT-Services, ist für uns gelebte Kultur und findet sich beispielsweise in Verträgen mit Partnern und Dienstleistern. Siemens achtet unter anderem darauf, dass bei Cloud Services erneuerbare Energien genutzt werden, dass bei der Auswahl von IT-Hardware die Reduktion des CO2-Fußabdrucks ein wichtiges Kriterium ist und auch, dass Altgeräte „refurbished“ werden. Diese Aktivitäten kombinieren wir mit der Unterstützung sozioökonomisch Benachteiligter durch Spenden von Geräten oder durch Kurse für Kinder und junge Heranwachsende, eine Hacker School oder auch Hackathons. Siemens beschäftigt 5300 Mitarbeitende mit Behinderung und unterstützt dies maßgeblich in allen Bereichen, so auch in der IT. Diversität bedeutet für mich, die Mitarbeitenden so zu akzeptieren und sie darin zu bestärken, so bei Siemens zu arbeiten, wie sie sind, unabhängig von ihrer kulturellen, religiösen oder sexuellen Orientierung – unser oberstes Gebot. Last but not least: Im „E“ für „Equity“ bei DEGREE ist das Ziel von 30 % Frauen in Führungsrollen im Topmanagement bis 2025 festgelegt. Darüber hinaus fördern wir mit speziellen Programmen wie in der Kooperation mit dem BDI, „SheTransformsIT“ oder auch mit Veranstaltungen wie „Female in Tech“ sehr aktiv Frauen in Tech-Berufen und wollen hier bewusst Vorbehalte und Vorurteile abbauen.

### Andreas Maier:

Ja, ökologische Aspekte sind bei Entscheidungen in der AXA sehr wichtig. Wir investieren auf der einen Seite massiv in Diversity und auf der anderen Seite auch aus ökologischen Gründen in eine Migration auf eine Public Cloud. Zudem sind im sog. IT-Target-Letter Nachhaltigkeitsziele enthalten.

### Michael Müller-Wünsch:

Wir haben jüngst die Position der TECH-Ambassadorin installiert, deren Aufgabe es ist, die interessante Technologie-Domäne für junge Menschen, insbesondere Frauen aber auch Quereinsteigerinnen und Quereinsteiger zu erklären. Wir bemühen uns mit Extra-Konferenzen, wie beispielsweise der develop<HER> genau diese Zielgruppe durch Fach-Workshops über die Vielfalt der Arbeitsfelder in der Technologie-Industrie zu informieren. Außerdem engagieren wir uns mit Vereinen, wie beispielsweise den ITgirls oder der Hackerschool, bei Schülerinnen und Schülern das Zukunftsfeld der Technologie-Berufe in die Aufmerksamkeit zu bringen. Dabei hilft sicherlich auch, dass wir uns sogar mit Forschungseinrichtungen darum bemühen, trotz der steigenden Digitalisierung bei uns und in der Gesellschaft, den damit verbundenen CO2-Footprint zu reduzieren. Eine Initiative ist der Daten- und Algorithmen-Minimalismus. Solche Programme inspirieren viele Menschen, bei OTTO zu arbeiten.

### Rolf Olmesdahl:

Nachhaltigkeit und Diversität haben bei Raiffeisen in den letzten Jahren massiv an Bedeutung gewonnen. Sämtliche IT-Beschaffungen werden auch in Bezug auf Nachhaltigkeitsaspekte beurteilt. Die Förderung von Frauen in der IT ist mit konkreten Zielsetzungen und einem umfassenden Maßnahmenpaket unterlegt.

### Ursula Soritsch-Renier:

Sustainability ist für Saint-Gobain ein zentrales Thema, das sehr viele Entscheidungen beeinflusst. Die Baubranche ist weltweit für 40 % des CO2-Ausstoßes verantwortlich. Das derzeitige Wachstum wird anhalten, da u. a. die Weltbevölkerung und auch die Urbanisierung weiterwachsen werden. Das größte Unternehmen der Branche, Saint-Gobain, ist hier gefordert, seinen Beitrag zu leisten. Wir tragen eine besondere Verantwortung und arbeiten an allen Fronten, um unseren CO2-Ausstoß zu reduzieren. Digitalisierung spielt neben den spezifischen Baumaterialien dabei eine große Rolle.

Genderfragen und Diversity sind natürlich in einem großen Unternehmen wie Saint-Gobain von wichtiger Bedeutung. Bei uns spiegelt sich dies bereits im Top Management wider. Der Vorstand besteht zu 50 % aus Nicht-Franzosen und der Frauenanteil liegt bei 38 %.

In der IT-Branche ist dieser Prozentsatz nur schwierig zu verwirklichen. Gibt es in Indien noch mehr in IT ausgebildete Frauen, sind es in Europa einfach zu wenige, welche diesen Weg beschreiten. Grundsätzlich glaube ich, dass es da um schlechte Public Relation geht. Wie viele Teenager wissen, dass es in der IT eine große Anzahl an unterschiedlichen und spannenden Berufen gibt? Das, was man unter „IT“ als Job versteht, nämlich Programmieren, macht vielleicht 10–20 % der eigentlichen Positionen aus. Ich würde mir mehr Kommunikation über die Vielfalt der Aufgaben innerhalb einer IT wünschen, damit sich dann mehr Frauen für eine solche Karriere entscheiden.

### Patrick Naef:

Bei der Rekrutierung von Führungskräften, wie CIOs, sehen wir heute sehr oft die Anforderung an Diversität. Beispielsweise verlangen heute einige Firmen einen gewissen Prozentsatz von Kandidatinnen auf der Shortlist. Jedoch ist Gender nur ein Aspekt der Diversity. Wir sehen die Anforderung an Diversität in der Altersstruktur, jedoch kaum bei der kulturellen Herkunft, geschweige denn bei der Ausbildung. Da sind die meisten Firmen noch sehr traditionell unterwegs. Vermutlich hängt das auch damit zusammen, dass Gender Diversity heute in aller Munde ist und jeder bei diesem Thema gut dastehen will, jedoch andere Dimensionen der Diversity in den Medien kaum erwähnt werden.

### *Walter Brenner:*

*Sustainability, Genderfragen und Diversity werden auch in der IT in den nächsten Jahren eine große und immer wichtigere Rolle spielen. Da sind sich alle CIOs, die an diesem Dialog teilnehmen, einig. Auf der einen Seite geht es darum, Antworten auf diese Herausforderungen innerhalb der IT-Organisation zu finden, auf der anderen Seite, vor allem in ökologischen Fragen, einen Beitrag für das ganze Unternehmen zu leisten.*

*Am Ende dieses Dialogs angekommen, danke ich Ihnen für Ihre Zeit und für die inspirierenden Antworten auf meine Fragen.*

## Epilog

Die CIOs, die für diesen Dialog gewonnen werden konnten, zeigen aus Sicht der Autorin und des Autors dieses Artikels eindrucksvoll, dass sie einen grossen, entscheidenden Beitrag zur Digitalisierung ihres Unternehmens geleistet haben und auch in Zukunft leisten wollen. Sie sind sich der Herausforderungen für den CIO durch den verstärkten Einsatz der Informations- und Kommunikationstechnik bewusst: Es gilt Innovation auf der einen Seite und einen sicheren unterbrechungsfreien Betrieb auf der anderen Seite zu verbinden. Diese „Schere“ ist nicht neu. Sie begleitet die CIOs schon seit vielen Jahren. Für die Zukunft spannend ist, wie die CIOs ihre Bereiche hinsichtlich der kommenden gesellschaftlichen Herausforderungen, wie beispielweise Nachhaltigkeit und Diversity, aufstellen. Die Statements der CIOs und des Headhunters zeigen deutlich, dass es auch in Zukunft spannende Fragen für die Wirtschaftsinformatik gibt, deren Bearbeitung sich in der Forschung lohnen wird.

## Footnotes

1 Unter dem „Basket of Eight“ werden die acht führenden Zeitschriften der Disziplin Information Systems verstanden. Siehe dazu https://aisnet.org/general/custom.asp?page=SeniorScholarBasket.

2 Das Gespräch mit Rolf Olmesdahl wurde Mitte November 2021 geführt.

3 Das Minimum Viable Product (MVP) beschreibt in der agilen Entwicklung das kleinste überlebensfähige Produkt, mit dem frühzeitig, unter realistischen Bedingungen, Feedback von Kundinnen und Kunden eingeholt werden soll.
